# Neurobehavioral and Oxidative Stress Effects of SiO_2_ Nanoparticles in Zebrafish and the Protective Role of N-Acetylcysteine

**DOI:** 10.3390/biomedicines13071762

**Published:** 2025-07-18

**Authors:** Viorica Rarinca, Irina-Luciana Gurzu, Mircea Nicusor Nicoara, Alin Ciobica, Malina Visternicu, Catalina Ionescu, Ioana Miruna Balmus, Gabriel-Ionut Plavan, Elena Todirascu-Ciornea, Bogdan Gurzu

**Affiliations:** 1 Doctoral School of Geosciences, Faculty of Geography and Geology, Alexandru Ioan Cuza University of Iași, Carol I Avenue, No 20A, 700505 Iasi, Romania; rarinca_viorica@yahoo.com (V.R.); mirmag@uaic.ro (M.N.N.); 2 Doctoral School of Biology, Faculty of Biology, Alexandru Ioan Cuza University of Iași, Carol I Avenue, 20A, 700505 Iasi, Romania; malina.visternicu@yahoo.ro; 3 Preclinical Department, Apollonia University, Pacurari Street 11, 700511 Iasi, Romania; alin.ciobica@uaic.ro; 4 Faculty of Medicine, University of Medicine and Pharmacy Grigore T. Popa, 700115 Iasi, Romania; 5 Faculty of Biology, Alexandru Ioan Cuza University of Iași, Carol I Avenue, 20A, 700505 Iasi, Romania; catalinaionescu81@yahoo.com (C.I.); gabriel.plavan@uaic.ro (G.-I.P.); ciornea@uaic.ro (E.T.-C.); 6 Department of Exact Sciences and Natural Sciences, Institute of Interdisciplinary Research, Alexandru Ioan Cuza University of Iasi, 700057 Iasi, Romania; balmus.ioanamiruna@yahoo.com; 7 Department of Morfofunctional Sciences, Faculty of Medicine, University of Medicine and Pharmacy Grigore T. Popa, 16th Universitatii Street, 700115 Iasi, Romania; bgurzu@yahoo.com

**Keywords:** silicon dioxide nanoparticles, zebrafish, oxidative stress, social test, color preference test, N-acetylcysteine

## Abstract

**Background/Objectives:** Silicon dioxide nanoparticles (SiO_2_NPs) do not exist in isolation in the environment but can interact with other substances, thus influencing their toxic effects on aquatic organisms. We assessed the combined impact of SiO_2_NPs and N-acetylcysteine (NAC), an antioxidant with the potential to counteract nanoparticle-induced oxidative stress (OS). **Methods**: Behavioral assessments, including the social interaction test and color preference test, were performed to evaluate neurobehavioral changes. OS biomarkers, including malondialdehyde (MDA) levels for lipid peroxidation and the activity of key antioxidant enzymes such as glutathione peroxidase (GPx), catalase (CAT), and superoxide dismutase (SOD), were assessed to evaluate the extent of cellular damage. **Results**: The results indicate that prolonged exposure to SiO_2_NPs induces significant behavioral disruptions, including reduced exploratory behavior and increased anxiety-like responses. Furthermore, biochemical analysis revealed increased OS, suggesting nanoparticle-induced cellular toxicity. NAC co-treatment partially reversed these effects, particularly improving locomotor outcomes and antioxidant response, but was less effective on social behavior. **Conclusions:** These findings highlight the ecological and health risks posed by SiO_2_NPs and point toward the need for further toxicological studies on their long-term biological effects.

## 1. Introduction

SiO_2_NPs represent a major class of nanomaterials, valued for their unique physicochemical and broad applicability across biomedical, industrial, and consumer fields [1,2]. Common synthesis approaches include the sol–gel method, which enables precise control over particle size and morphology, and green synthesis, which utilizes plant extracts to enhance biocompatibility and stability through the incorporation of natural phytochemicals [3,4,5,6].

Due to their widespread use, human exposure to SiO_2_NPs has become increasingly common, occurring through inhalation, ingestion, dermal contact, and parenteral routes. Their physicochemical properties, such as size and surface chemistry, play a pivotal role in determining their toxicological impact [7]. These factors must be carefully considered when assessing potential health risks associated with SiO_2_NPs exposure.

Cellular uptake of SiO_2_NPs varies depending on particle size and cell type but commonly results in OS, cytotoxicity, apoptosis, and inflammatory responses [8]. Their ability to cross physiological barriers and induce OS, DNA damage, upregulation of apoptosis markers, and reproductive toxicity raises important health concerns that require careful assessment [1,9,10,11]. As a result, SiO_2_NPs can affect multiple organs [11], not only the brain [8], lungs [12], skin [13], and liver [4].

Animal studies reveal that while low doses may not induce severe acute toxicity, the long-term effects and mechanisms of action require further investigation. The variability in findings underscores the need for standardized testing protocols to better assess the risks associated with SiO_2_NP exposure across different environments and populations.

Despite recent advances in the understanding of anxiety disorders, existing pharmacological treatments often have limited efficacy and significant side effects. A promising approach to improving treatment options is drug repurposing, which involves finding new uses for established drugs. NAC, traditionally used as a mucolytic agent and paracetamol antidote, has gained attention for its potential in treating various neuropsychiatric disorders due to its complex mechanisms involving antioxidant, inflammatory, neurotrophic, and glutamate pathways [14]. In addition, it has been shown to exert multi-target effects through neuroprotective, antioxidant, and neurotrophic mechanisms, as well as functioning as a glutamate modulator [15].

In a study evaluating the effects of NAC on anxiety-related behaviors in zebrafish, it was found that NAC did not significantly change behaviors in the novel tank test but effectively prevented anxiety-like behaviors triggered by acute stressors. In addition, NAC increased the time zebrafish spent in lighted areas during light/dark tests, suggesting an anti-stress effect [16]. The genetic similarities between zebrafish and mammals lend translational validity to these results, supporting further clinical trials to explore the potential of NAC in treating anxiety disorders [15]. In another study, zebrafish were pretreated with NAC (10 mg/L for 10 min) before being exposed to either standard tank water (EtOH−) or 1% EtOH (EtOH+) for 60 min. Thus, zebrafish exposed to EtOH were observed to have reduced locomotor activity. In addition, ethanol exposure resulted in oxidative damage, indicated by increased lipid peroxidation, decreased non-protein thiols, and increased production of ROS. NAC treatment effectively prevented both behavioral changes and OS in EtOH-exposed zebrafish [17].

The environmental risks associated with SiO_2_NPs are significant. Their small size allows for easy penetration into biological systems. These nanoparticles are capable of inducing OS, inflammation, and neurobehavioral effects, affecting human health and aquatic life. While SiO_2_NPs offer numerous benefits in various applications, their toxicity and environmental impact cannot be overlooked.

In this context, NAC is known for its antioxidant and neuroprotective properties [18]. However, a significant knowledge gap remains regarding the effectiveness of NAC in counteracting the specific toxic effects induced by SiO_2_NPs, especially in aquatic organisms. This study aims to evaluate the interaction between SiO_2_NP exposure and NAC treatment, focusing on behavioral and biochemical effects in zebrafish. This research will help clarify the risks posed by these nanoparticles and the potential protective role of NAC. Therefore, this study aims to investigate whether NAC can mitigate the behavioral and biochemical alterations induced by SiO_2_NP exposure in zebrafish.

## 2. Materials and Methods

### 2.1. Zebrafish

**Figure 1:**
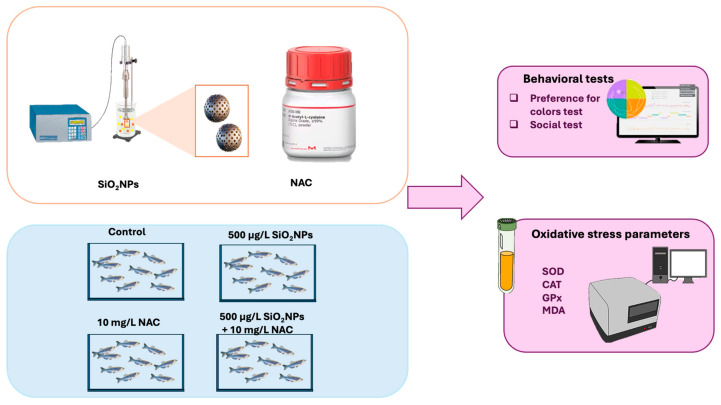
Experimental design illustrating zebrafish exposure to SiO_2_NPs and NAC, followed by behavioral, immunological, and oxidative stress assessments.

Wild-type AB strain zebrafish (6–8 months old) with a balanced sex ratio (1:1, male to female), were housed under standard laboratory conditions, following a 14:10 light/dark cycle, and maintained at 28 °C with pH 7.2 and a conductance range of 470–520 μS, according to established protocols [19]. For this experiment (Figure 1), adult zebrafish were maintained in 5 L experimental aquaria, fed TetraMin flakes (Tetra GmbH, Melle, Germany) twice daily, and acclimated to experimental conditions for a week prior to the study. All experiments involving the use of zebrafish were carried out in accordance with the EU Commission Recommendation (2007), Directive 2010/63/EU of the European Parliament and of the Council of 22 September 2010 on guidelines for housing, care and the protection of animals used for experimental purposes [20]. The experiments were conducted in accordance with the Declaration of Helsinki, as well as the legislation of Romania and the European Union regarding the use of animals in biomedical research. Also, all experiments and procedures were conducted with the approval of the Ethics Committee of the Faculty of Biology (“Alexandru Ioan Cuza” University in Iasi). All procedures were performed by limiting the number of individuals, according to the ARRIVE guidelines [21]. At the end of the experiment, zebrafish were euthanized by rapid cooling in ice-cold water (2–4 °C) for 10 min. This method is commonly used for small fish and minimizes distress [22].

### 2.2. Materials

Silicon dioxide nanoparticles (SiO_2_NPs, CAS number 7631-86-9, particle size 50 nm) and N-acetylcysteine (NAC, CAS number 616-91-1) were acquired from Sigma-Aldrich (St. Louis, MO, USA). The SiO_2_NPs suspension was sonicated for 30 min before use to ensure uniform dispersion. NAC was freshly prepared in deionized water at a concentration of 10 mg/L before exposure. The concentrations were chosen based on previous studies to reflect realistic environmental and pharmacological conditions for zebrafish exposure. The concentrations were selected based on previous studies demonstrating neurobehavioral and oxidative effects in zebrafish and are within the experimental ranges commonly used in toxicological evaluations, though they exceed predicted environmental concentrations [14,15,16,23].

### 2.3. Zebrafish Exposed to SiO_2_NPs and NAC

#### 2.3.1. Experimental Setup for Behavioral Testing

Social interaction and color preference behavior of zebrafish, previously exposed to SiO_2_NPs for 7 days and then treated with NAC, were observed in the T-maze, following the protocol described by previous work [24,25]. The experimental apparatus consisted of three arms, left, right, and central (10 × 10 × 10 cm), with a camera positioned above for recording the behavior. For the color preference test, the maze was modified by coloring the right arm green and the left arm red. For the social interaction test, one arm of the maze contained a transparent compartment with conspecifics, while the other arm remained empty. This setup allowed for the measurement of social preference behavior based on the zebrafish’s choice of arm.

#### 2.3.2. Acclimatization for Preference Tests

Acclimatization in the three-arm occurred over several days. To ensure familiarity and reduce novelty-induced stress. During this period, all fish in the experimental group were placed in the maze for gradually decreasing durations. On the first day, the entire group of adult zebrafish was placed in the maze on the first day and allowed to swim for 20 min. Then, on each subsequent day, the number of fish and exposure times were gradually reduced (half the group for 10 min, three to four fish for 8 min, two to three fish for 5 min, one to two fish for 5 min, and finally a single fish for 5 min) [26]. This progressive reduction was intended to minimize anxiety, novelty stress, and social isolation effects during testing, and to allow the animals to gradually habituate to individual exploration of the maze. Acclimatization was assessed by monitoring swimming activity: fish that remained immobile or displayed frequent freezing behavior were excluded from further testing.

#### 2.3.3. Behavioral Parameters for Color Preference

Total distance traveled (cm) was used as an indicator of general locomotor activity and spatial exploration. Higher values reflect increased activity and exploratory behavior, while reduced distance may suggest anxiety-like responses or motor disorders. Speed (cm/s) represents the overall swimming speed of the zebrafish and provides insight into their emotional state; decreased speed may indicate stress or anxiety.

The time spent in the left (s) and right arms (s) of the apparatus during the 5 min test was recorded separately. A longer duration in one of the arms may indicate a preference for that side or a color-based attraction, while reduced time may suggest avoidance behavior possibly related to anxiety. Additionally, research has shown that zebrafish prefer the color red over green, a preference that is associated with easier detection of longer wavelength colors in the aquatic environment, as well as the possible association of red with food sources such as zooplankton or microcrustaceans containing carotenoids [27].

The cumulative duration (s) of mobility refers to the total time the fish were actively moving, reflecting overall activity levels. In contrast, the cumulative duration (s) of immobility corresponds to periods of inactivity, which can be interpreted as freezing behavior, commonly associated with anxiety-like states in zebrafish.

Additionally, rotational behavior was measured in both clockwise and counterclockwise directions. Repetitive circles or asymmetrical rotational patterns may suggest changes in motor coordination or increased levels of anxiety.

#### 2.3.4. Behavioral Parameters for Social Test

For the social interaction test, specific behavioral parameters were analyzed to evaluate the social behavior of zebrafish. One of the main parameters assessed was the total distance traveled (cm), which serves as an indicator of general locomotor activity and willingness to explore the environment, including the area near conspecifics. A higher distance may indicate increased exploratory and social interest, while reduced distance can suggest avoidance or anxiety.

Velocity (cm/s) was also recorded as a measure of swimming speed and general activity. Lower values could reflect anxiety-like states or social withdrawal, while higher velocity may indicate an active engagement with the environment and conspecifics.

The time spent in the left and right arms of the testing apparatus was measured, with one of the arms containing a transparent compartment with conspecifics, and the other remaining empty. A longer duration spent in the arm with conspecifics may suggest social preference and attraction, whereas a preference for the empty arm could indicate social avoidance or stress.

In addition, acceleration (cm/s^2^) was monitored to assess changes in movement dynamics. Variability in acceleration can reflect shifts in emotional arousal or motivational states during social encounters.

### 2.4. Preparation of Homogenates and Biochemical Parameter Analysis

Parameter Analysis Enzyme activity was assessed at a single time point, after 7 days of continuous exposure to SiO_2_NPs and/or NAC. This approach was designed to capture the cumulative oxidative stress response associated with subacute exposure to the toxin. Immediately following the final behavioral test, the animals were individually placed in a glass dish containing ice-cold water (2–4 °C) for 10 min, and then rapidly euthanized, as previously described by Jorge et al. [28]. The next day, fish in the same batch were individually weighed (~3–9 mg) and homogenized in an extraction phosphate buffer solution (0.1 M potassium phosphate buffer, pH 7.4 with 1.15% KCl) at a 1:10 ratio (weight/volume). The homogenate was centrifuged (15 min at 3500 rpm), and the supernatant was then used for biochemical parameter determination.

#### 2.4.1. Superoxide Dismutase Activity Determination

In this study, superoxide dismutase activity was determined following the protocol described by Winterbourn et al. [29]. Briefly, the ability of the enzyme to inhibit the reduction of nitroblue tetrazolium (NBT) by superoxide free radicals, generated by riboflavin photoreduction, was monitored. The final mixture included 0.067 M potassium phosphate solution, enzyme extract, 0.1 M EDTA, 0.12 mM riboflavin, and 1.5 mM NBT solution. Samples were read at 560 nm. SOD-specific activity was calculated in enzymatic units per mg of protein, determined using the Bradford method [30].

#### 2.4.2. Catalase Activity Determination

CAT activity was measured using a simple colorimetric method initially described by Sinha [31]. For each sample, 125 µL of enzyme homogenate and 125 µL of 0.16 M H_2_O_2_ solution were mixed. After 3 min, the reaction was halted by adding 500 µL of potassium dichromate–glacial acetic acid solution, and the tubes were incubated at 95 °C for 10 min. After centrifugation at 14,000 rpm for 5 min, the absorbance of the supernatant was read at 570 nm. Enzyme activity was expressed in µmoles of H_2_O_2_ consumed/min/mg protein.

#### 2.4.3. Glutathione Peroxidase Activity Determination

GPx activity was evaluated using the protocol described by Fukuzawa and Tokumura [32]. This method is based on the enzyme’s ability to catalyze the decomposition of H_2_O_2_ with GSH as the reductant, producing oxidized glutathione (G-S-S-G) and water. To each sample, 78 µL of enzyme extract, 475 µL of 0.25 M sodium phosphate buffer (pH 7.4), 36 µL of 25 mM EDTA, and 36 µL of 0.4 M NaN3 solution were added. After 10 min of incubation at 37 °C, 50 µL of 50 mM GSH solution and 36 µL of 50 mM H_2_O_2_ solution were added. The reaction was stopped with 730 µL of 7% metaphosphoric acid, and the samples were centrifuged for 10 min at 14,000 rpm. A 100 µL aliquot of supernatant was transferred to a new tube, and 1270 µL of 0.3 M disodium phosphate solution and 136 µL of 0.04% DTNB solution were added. After 10 min, the absorbance was measured at 412 nm. The specific GPx activity was calculated based on the enzymatic units per mg protein, as determined by the Bradford method [30].

#### 2.4.4. Malondialdehyde Level Determination

The malondialdehyde content, as a marker of lipid peroxidation, was determined using the method described by Ohkawa et al. [33], with slight modifications. Briefly, 200 µL of enzyme extract was mixed with 1 mL of 20% trichloroacetic acid (TCA) and 1 mL of 0.67% thiobarbituric acid (TBA) solution. The mixture was then heated in a boiling water bath at 95 °C for 30 min. After cooling on ice, the samples were centrifuged at 3000 rpm for 10 min. The absorbance of the supernatant was measured at 532 nm using a spectrophotometer Specord 210 Plus (Analytik Jena, Jena, Germany). MDA concentration was calculated using an extinction coefficient of 1.56 × 10^5^ M^−1^cm^−1^ and expressed as nmol MDA/mg protein.

### 2.5. Statistical Analysis

To assess whether the data followed a normal distribution, we applied the Shapiro–Wilk test using GraphPad Prism 9 (GraphPad Software, San Diego, CA, USA). Potential outliers were identified using Grubbs’ test and removed where appropriate. Based on the results of the normality assessment, data were analyzed using one-way ANOVA followed by Tukey’s post hoc test for multiple comparisons. Results are expressed as mean ± standard deviation (SD), and a *p*-value < 0.05 was considered statistically significant.

## 3. Results

### 3.1. Social Test

**Figure 2:**
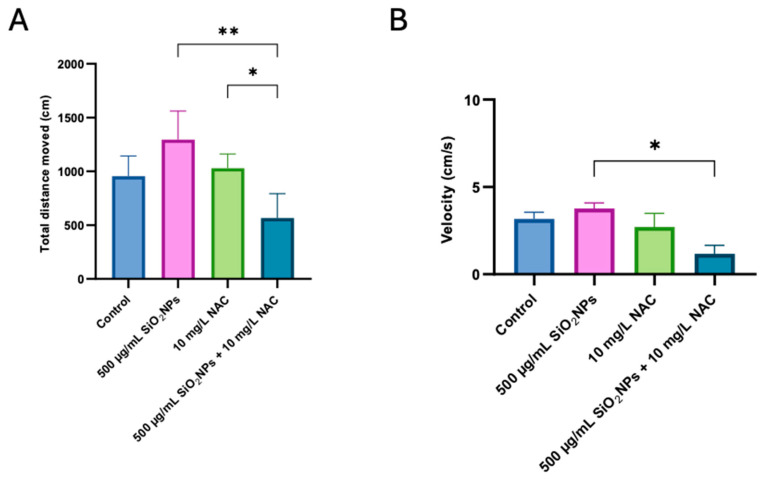
Effects of SiO_2_NPs and NAC on the social preference of zebrafish in the T-maze test. (**A**) Total distance moved (cm). (**B**) Velocity (cm/s). The experimental groups include control, 500 µg/mL SiO_2_NPs, 10 mg/L NAC, and the combined administration of 500 µg/mL SiO_2_NPs and 10 mg/L NAC. Data are presented as mean ± standard deviation (SD) (*n* = 5/group). Statistical significance was assessed by ANOVA followed by Tukey’s correction for multiple comparisons. Asterisks indicate significance levels: * *p* < 0.05, ** *p* < 0.01.

Zebrafish treated with SiO_2_NPs and NAC at different concentrations were transferred to a T-maze for social behavior testing. To ensure consistent and reliable behavioral responses, testing was conducted between 10 a.m. and 4 p.m., in line with the recommendations of Tagkalidou et al., who advised that this timeframe corresponds with the most stable behavioral patterns in zebrafish larvae and minimizes variability due to circadian rhythms [34]. The results of the zebrafish social test are shown in Figure 2.

Figure 2A illustrates the total distance moved by zebrafish following exposure to SiO_2_NPs and/or NAC treatment. Although there was no significant difference in locomotor activity between the control group and any of the other treatment groups, a significant increase in activity was observed in zebrafish exposed to 500 μg/mL SiO_2_NPs or NAC alone, compared to the combined SiO_2_NPs + NAC treatment. These results suggest a potential modulatory or protective effect of NAC when co-administered with SiO_2_NPs, reducing the hyperactivity observed with single treatments.

In Figure 2B, exposure to 500 μg/mL SiO_2_NPs and NAC decreased velocity compared to the group where the nanoparticles were administered individually. This suggests a potential interactive effect between NAC and SiO_2_NPs on locomotor function. While NAC is generally considered to have protective properties, its co-administration may alter the physiological response to nanoparticles in ways that are not purely beneficial. The reduced velocity might reflect an additive or even synergistic impact on neuromotor pathways, possibly due to metabolic interference, increased sedation, or modulation of neurotransmitter systems involved in movement regulation. Further investigation is needed to clarify whether this reduction is a result of enhanced toxicity, compensatory behavioral adaptation, or pharmacodynamic interaction between NAC and the nanoparticles.

**Figure 3:**
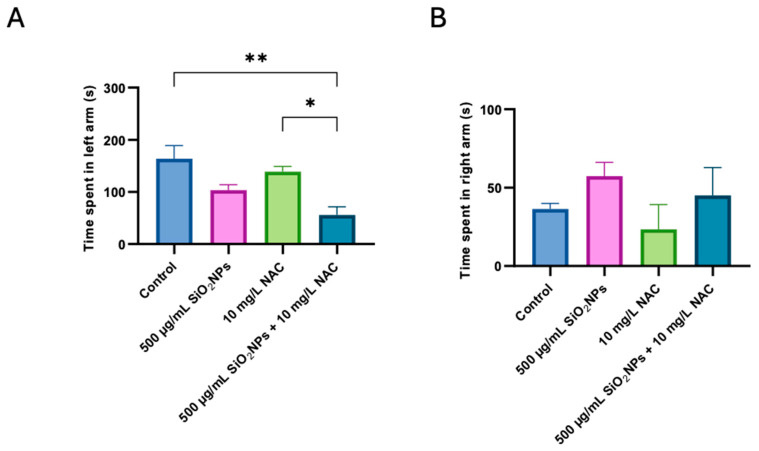
Effects of SiO_2_NPs and NAC on the social preference of zebrafish in the T-maze test. (**A**) Time spent in left arm (s). (**B**) Time spent in right arm (s). The experimental groups include control, 500 µg/mL SiO_2_NPs, 10 mg/L NAC, and the combined administration of 500 µg/mL SiO_2_NPs and 10 mg/L NAC. Data are presented as mean ± standard deviation (SD) (*n* = 5/group). Statistical significance was assessed by ANOVA followed by Tukey’s correction for multiple comparisons. Asterisks indicate significance levels: * *p* < 0.05, ** *p* < 0.01.

Figure 3A shows that zebrafish exposed to 500 µg/mL SiO_2_NPs spent less time in the social stimulus arm. However, these decreases were not statistically significant. As for the group exposed to NAC alone, it did not have a significant effect compared to the Control group. In contrast, the SiO_2_NPs + NAC group has spent the least time in the social area, suggesting a possible negative interaction between SiO_2_ nanoparticles and NAC, which could affect social behavior more than any individually administered treatment. This observation indicates that, although NAC does not have a significant impact on its own, its combination with SiO_2_NPs can amplify the adverse effects at the behavioral level.

Figure 3B indicates no significant difference during the time spent in the right arm of the maze between groups, indicating that general spatial exploration remained unaffected across conditions.

### 3.2. Preference for Colors Test

**Figure 4:**
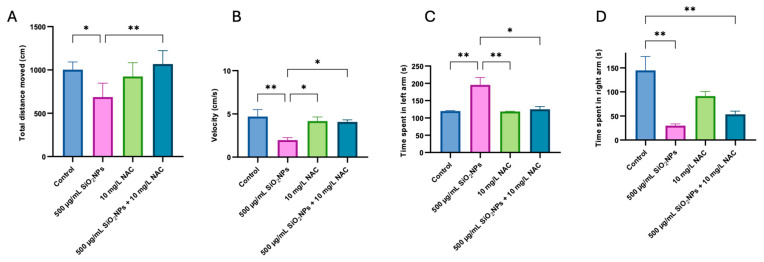
Effects of SiO_2_NP and NAC administration on total distance moved (**A**), velocity (**B**), time spent in left arm (s) (**C**), and time spent in right arm (s) (**D**) in zebrafish during the color preference test. The experimental groups include control, 500 µg/mL SiO_2_NPs, 10 mg/L NAC, and the combined administration of 500 µg/mL SiO_2_NPs and 10 mg/L NAC. Data are presented as mean ± standard deviation (SD) (*n* = 5/group). Statistical significance was assessed by ANOVA followed by Tukey’s correction for multiple comparisons. Asterisks indicate significance levels: * *p* < 0.05, ** *p* < 0.01.

In Figure 4A, zebrafish exposed to 500 μg/mL SiO_2_NPs showed a significant reduction in total distance moved compared to the control group (*p* = 0.013). This suggests impaired locomotor activity, potentially due to neurotoxicity or stress-induced hypoactivity. Co-treatment with NAC significantly increased this parameter (*p* = 0.002) compared to the group where only individual SiO_2_NPs were administered, which suggests a protective effect in restoring normal movement.

Figure 4B shows reduced swimming speed in the 500 µg/mL SiO_2_NPs group compared to the control group (*p* = 0.047), reinforcing the observation of SiO_2_NP-induced behavioral suppression. NAC treatment prevented this decrease, returning speed to control levels, further supporting its neuroprotective potential.

Figure 4C shows that SiO_2_NPs increased time spent in the red arm compared to the control group (*p* = 0.009), which may reflect increased anxiety-like behavior or altered environmental preference. NAC co-treatment reversed this effect, observing a significant difference compared to the SiO_2_NPs group (*p* = 0.008), suggesting a modulatory role in affective behavior.

In Figure 4D, the administration of 500 µg/mL SiO_2_NPs caused a significant decrease in time spent in the green arm (*p* = 0.001). NAC partially reversed this effect (*p* = 0.010), though not to control levels.

**Figure 5:**
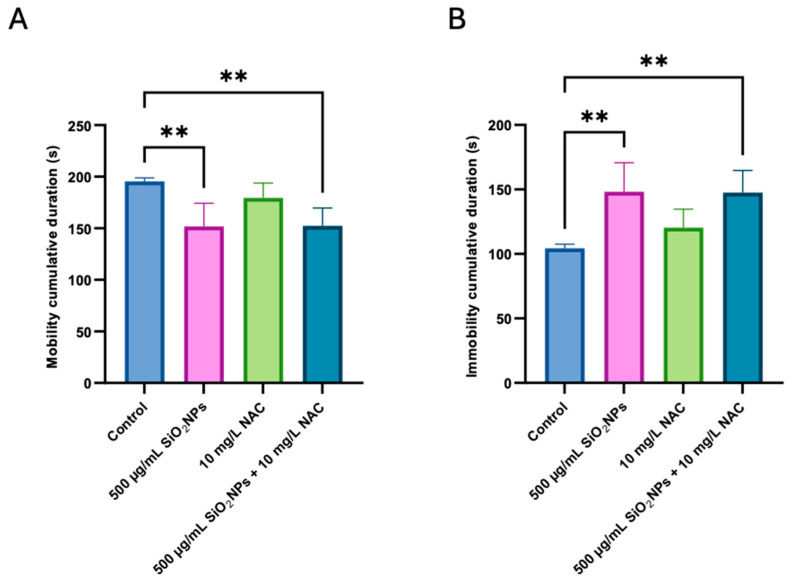
Effects of SiO_2_NP and NAC administration on mobility cumulative duration (s) (**A**), and immobility cumulative duration (s) (**B**) The experimental groups include control, 500 µg/mL SiO_2_NPs, 10 mg/L NAC, and the combined administration of 500 µg/mL SiO_2_NPs and 10 mg/L NAC. Data are presented as mean ± standard deviation (SD) (*n* = 5/group). Statistical significance was assessed by ANOVA followed by Tukey’s correction for multiple comparisons. Asterisks indicate significance levels: ** *p* < 0.01.

Figure 5A shows a significant reduction in mobility cumulative duration in groups treated with 500 µg/mL SiO_2_NPs alone (*p* = 0.0072) or with NAC (*p* = 0.0082) compared to the control group. NAC treatment alone maintained mobility near control levels.

Regarding the immobility cumulative duration, there is a significant increase in both 500 µg/mL SiO_2_NPs and SiO_2_NPs + NAC groups compared to the control (*p* = 0.0072 and *p* = 0.0082, respectively).

These results collectively suggest that SiO_2_NPs induce significant behavioral deficits in zebrafish, particularly affecting locomotion and color preference, with NAC exerting a substantial, though not complete, protective effect.

### 3.3. Oxidative Stress

**Figure 6:**
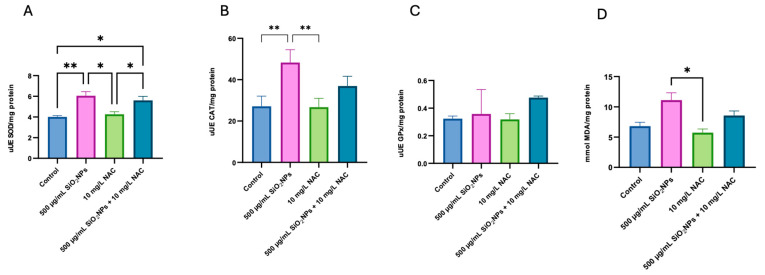
Levels of oxidative stress markers in zebrafish tissue after exposure to 500 µg/mL SiO_2_ nanoparticles, 10 mg/L N-acetylcysteine, and their combination (SiO_2_NPs 500 µg/mL + NAC 10 mg/L). (**A**) Superoxide dismutase (SOD), (**B**) catalase (CAT), (**C**) glutathione peroxidase (GPx), and (**D**) malondialdehyde (MDA). Data are expressed as mean ± standard deviation (SD) (*n* = 3/group). Statistical significance was assessed by one-way ANOVA followed by Tukey’s *post hoc* test. Asterisks indicate significance levels: * *p* < 0.05, ** *p* < 0.01.

Figure 6A shows a significant increase in SOD activity in the group treated with individually administered SiO_2_NPs, suggesting an accentuated oxidative response. Although the group exposed to a concentration of 500 µg/mL presented lower activity compared to the other doses, it was, however, significantly increased compared to the control (*p* = 0.0175), suggesting a stress-adaptive response. In contrast, in the group treated concomitantly with NAC and SiO_2_NPs, the SOD activity did not differ significantly from the individually administered SiO_2_NPs group or the control group, indicating that NAC may attenuate the oxidative response triggered by SiO_2_NP exposure without overstimulating SOD production.

Figure 6B shows a significant increase in CAT activity in the group treated with SiO_2_NPs compared with the control (*p* = 0.004), which supports the activation of endogenous antioxidant mechanisms. Interestingly, the group that received the combined treatment NAC + SiO_2_NPs presented reduced activity compared to the group treated only with nanoparticles, suggesting a possible inhibition or normalization of the antioxidant response in the presence of NAC.

Figure 6C did not highlight significant differences in glutathione peroxidase activity among the analyzed groups. This lack of variation may indicate that GPx activity remained stable under the given exposure conditions, suggesting that this specific antioxidant pathway was not markedly affected by SiO_2_NPs or NAC treatment.

Figure 6D shows that levels of malondialdehyde, a marker of lipid peroxidation, do not differ significantly between groups. This suggests that the level of oxidative stress induced by SiO_2_NP exposure may not have been sufficient to cause measurable lipid membrane damage under the experimental conditions.

## 4. Discussion

Zebrafish are an increasingly valuable model in behavioral neuroscience due to their advanced visual system, which includes tetrachromatic vision capable of detecting red, green, blue, and ultraviolet light. They exhibit consistent innate color preferences, typically favoring red and green over yellow and blue [35], which are closely tied to cognitive and emotional states. These preferences are sensitive to neurochemical and environmental alterations, making them a reliable indicator in neurobehavioral assessments.

In this study, exposure to SiO_2_NPs disrupted color preference behavior. Zebrafish displayed a significantly increased preference for the red compartment (Figure 4C, *p* = 0.009) and a marked aversion to green (Figure 4D, *p* = 0.001), which deviates from the expected red > green > blue > yellow pattern. These shifts suggest that SiO_2_NP-induced oxidative stress (OS) and neurotoxicity impair sensory integration and emotional processing. Elevated antioxidant enzyme activity (SOD, *p* = 0.0175, Figure 6A, and CAT, *p* = 0.004, Figure 6B) confirmed a physiological stress response, although MDA levels did not show statistically significant changes.

These alterations align with prior research showing that oxidative stress disrupts dopaminergic and serotonergic pathways, critical for reward-based decision making and sensory processing [36]. Moreover, the increase in immobility and reduced mobility (Figure 5A, *p* = 0.0072) observed in this study may reflect anxiety-like or stress-induced freezing behaviors, affecting color exploration and preference.

Co-treatment with NAC significantly mitigated many behavioral changes, including restoring time spent in the green compartment (Figure 4D, *p* = 0.010) and improving swimming speed and distance (Figure 4A,B). Our results confirm NAC’s antioxidant and neuroprotective role, consistent with its established effects in other zebrafish models of stress and neurotoxicity [17,18].

The behavioral and biochemical results from this study highlight the neurotoxic potential of SiO_2_NPs and the modulatory role of NAC. Exposure to SiO_2_NPs significantly altered zebrafish behavior in both social and color preference tasks, with distinct effects depending on the dose. Notably, NAC exhibited a partial protective effect, especially in locomotor and OS parameters.

SiO_2_NP exposure significantly reduced swimming speed and distance moved in the color preference test (Figure 4A,B, *p* = 0.013 and *p* = 0.047, respectively). However, co-exposure to SiO_2_NPs and NAC resulted in a significant reduction in total distance traveled and swimming speed compared to the animals where the nanoparticles were administered individually (Figure 2A,B). These findings suggest a potential interaction between SiO_2_NPs and NAC that may influence the locomotor behavior of zebrafish, highlighting the need for further investigation into their combined effects. NAC treatment normalized these parameters, confirming its antioxidant and neuroprotective properties (Figure 4A,B). Our findings are consistent with the complex locomotor response of zebrafish larvae exposed to SiO_2_NPs, as described by Shen et al. (2025) [37], who observed altered locomotion and anxiety-like behaviors following nanoparticle exposure. On the other hand, data from the literature support the idea that NAC may act as a protective agent for the neuromotor system, mainly by alleviating oxidative stress [38]. The reduction in total distance observed in the SiO_2_NP + NAC group, compared to either treatment alone, may suggest a potential modulatory or protective effect of NAC revealed when co-administered with SiO_2_NPs. Interestingly, NAC co-treatment restored swimming parameters to control (*p* < 0.05) levels in the color preference test, suggesting effective behavioral recovery.

In contrast, time spent in the social arm decreased following SiO_2_NP exposure (Figure 3A), indicative of reduced sociability or social avoidance. Although NAC alone showed a slight, non-significant tendency toward facilitating social interactions, its combination with SiO_2_NPs led to a further reduction in the time spent in the social arm (Figure 3A) compared to both the NAC-only and control groups. This suggests that while NAC may alleviate OS and motor hyperactivity, it does not fully reverse, and may even exacerbate, social behavior deficits when combined with SiO_2_NPs. These deficits could involve other mechanisms, such as disrupted neurotransmitter systems or neuroinflammation. The NAC-only group showed no statistically significant differences in social preference behavior compared to the control or the SiO_2_NPs-exposed groups (Figure 3A). While slight numerical variations were observed in the raw data, these fell within the range of normal biological variability and do not support any meaningful enhancement in sociability. Our findings do not support a pro-social effect of NAC when administered alone. Rather, our focus is on the relative outcomes and the influence of NAC on behavioral alterations induced by SiO_2_NPs exposure. This clarification helps prevent misinterpretation of the results and avoids overstating the behavioral effects of NAC.

Color preference behavior was also affected, with SiO_2_NPs administration increasing preference for the red compartment (Figure 4C, *p* = 0.009) and decreasing time spent in the green zone (Figure 4D, *p* = 0.001), both statistically significant. These changes may indicate altered visual perception, anxiety, or emotional processing. SiO_2_NPs also pose risks to aquatic ecosystems. Chronic exposure to these nanoparticles has been linked to detrimental effects on the reproductive performance of zebrafish, including reduced gonad weight and lower larval survival rates. Furthermore, changes in biochemical parameters such as cholesterol and protein levels were observed in exposed fish, indicating systemic toxicity [39]. In studies involving invertebrates such as water fleas (*Daphnia magna*), exposure to SiO_2_NPs led to physiological changes such as reduced swimming performance at sublethal concentrations [40], suggesting that even low doses of SiO_2_NPs can affect the health and behavior of aquatic organisms.

Although some research on strains that have been grown in the lab has indicated that zebrafish are attracted to colors with shorter wavelengths, such as blue and green, and avoid colors with longer wavelengths, such as red and yellow, other studies showed the opposite trend: zebrafish significantly avoided blue, but were attracted to shades of green, red, and yellow [27].

In a study, a domesticated zebrafish population and three wild-caught populations were used to examine whether there are differences in preferences for blue versus green and red versus green and how this affects learning [27]. Thus, the researchers noticed that green integrates well into the natural environment, while green and red present a greater opposition, with red being associated with the shades of food, and blue and green are colors with less opposition [41].

NAC treatment reversed these preferences, particularly restoring interest in the green compartment, suggesting the normalization of sensory or emotional integration. However, immobility duration increased significantly in the SiO_2_NPs + NAC group, suggesting that NAC may not fully counteract stress-induced freezing, and under certain conditions, could potentially exacerbate immobility.

Interestingly, although NAC co-treatment reversed several locomotor and color preference alterations induced by SiO_2_NPs, it appeared to exacerbate social avoidance in the T-maze task (Figure 3A). This divergence may reflect the involvement of distinct neural circuits regulating social versus exploratory behaviors. Since oxidative stress markers alone did not fully explain this behavioral pattern, additional mechanisms such as neuroinflammatory signaling, neurotransmitter dysregulation, or microglial activation may have contributed to the observed social deficits. Previous studies have demonstrated that silica nanoparticles can trigger inflammatory cascades and alter synaptic signaling even in the absence of marked oxidative damage [42]. While NAC is generally neuroprotective, its role as a glutamate modulator may differentially affect social behavior compared to locomotor or anxiety-related responses. Nonetheless, this study did not assess cytokine levels, neurotransmitter concentrations, or neuroinflammatory markers through immunohistochemistry. Future research incorporating these molecular and cellular endpoints will be crucial to determine whether the co-exposure to NAC and SiO_2_NPs results in synergistic or maladaptive effects across specific behavioral domains.

The OS data support the behavioral findings. SiO_2_NPs elevated SOD (*p* = 0.0175, Figure 6A) and CAT (*p* = 0.004, Figure 6B) activities, confirming the presence of oxidative stress. However, at 500 µg/mL, this response plateaued or declined, pointing to enzymatic exhaustion or damage. NAC treatment modulated SOD and CAT activities and tended to reduce MDA levels (Figure 6D), though not significantly, indicating partial oxidative stress mitigation. These effects likely underpin the protective behavioral outcomes observed.

GPx activity did not show significant differences between the SiO_2_NPs-treated and control groups (Figure 6C), suggesting that this antioxidant marker was not significantly affected by exposure. However, NAC treatment maintained GPx activity at levels close to those in the control group, highlighting a potential stabilizing effect on redox homeostasis.

MDA levels showed no significant differences among groups (Figure 6D), suggesting lipid damage was limited or compensated by antioxidant defenses. NAC and its co-administration reduced MDA levels, suggesting effective prevention of lipid membrane damage. Still, the intermediate MDA values in the NAC + SiO_2_NPs group suggest that while NAC attenuates OS, complete protection may not occur, especially under intense nanoparticle challenge.

In summary, SiO_2_NPs induce dose-dependent behavioral and oxidative changes in zebrafish, reflecting both neurostimulatory and neurotoxic effects. NAC provides substantial but incomplete protection, particularly evident in oxidative biomarkers (Figure 6A–D) and locomotor parameters (Figure 2A,B and Figure 4B–D). Its limited effect on social behavior (Figure 4A) and immobility (Figure 4A) suggests that additional pathways are involved, possibly beyond redox imbalance. Future studies should investigate synergistic treatments or alternative antioxidant strategies that target both oxidative and neurochemical pathways implicated in nanoparticle-induced toxicity.

A further limitation of our study concerns the concentration of SiO_2_NPs used. The selected dose of 500 µg/mL, although based on prior studies demonstrating behavioral and oxidative stress effects in adult zebrafish [23,43], substantially exceeds predicted environmental concentrations. Book and Backhaus, 2022, estimated a hazardous concentration (HC_05_) of 130 µg/L and a predicted no-effect concentration (PNEC) of 30 µg/L for aquatic species, suggesting that our model does not reflect environmentally realistic exposure levels [44]. While our chosen dose remains well below reported LC_50_ values [45], it should be interpreted as part of a high-dose modeling approach intended to reveal mechanistic insights and evaluate the efficacy of protective interventions. However, a wide variation in SiO_2_NP concentrations was reported across studies. For instance, studies have employed concentrations as low as 100 μg/mL for in vitro assessments, with higher doses (up to 20 mg/kg) used in vivo studies [46,47,48]. This strategy is consistent with previous toxicological research, including embryotoxicity studies that employed concentrations as high as 1100 to 4400 µg/mL [49], and behavioral studies in adults using doses between 100 and 1000 µg/mL. Nevertheless, future investigations should incorporate a broader range of concentrations to enhance environmental relevance and better simulate real-world exposure conditions. Additionally, exploring the dose-response relationship between SiO_2_NPs and NAC, including varying exposure ratios, may uncover important dynamics regarding the threshold and effectiveness of antioxidant protection across different behavioral and biochemical endpoints.

Another limitation of this study is the absence of mechanistic investigations at the molecular level. Although we evaluated behavioral alterations (Figure 2, Figure 3 and Figure 4) and oxidative stress biomarkers (Figure 6) to characterize the impact of SiO_2_NPs and the protective role of NAC, this study did not include analyses of gene or protein expression, neuroinflammatory pathways, or synaptic signaling. These mechanistic endpoints would provide a deeper understanding of the biological processes underlying the observed effects. Future work should employ molecular techniques such as quantitative polymerase chain reaction (PCR), enzyme-linked immunosorbent assay (ELISA), immunohistochemistry, or transcriptomic profiling to identify specific cellular pathways affected by nanoparticle exposure and to clarify the molecular mechanisms of NAC-mediated neuroprotection. Incorporating these methods will strengthen the translational relevance of the zebrafish model and improve our capacity to identify targets for intervention.

An additional limitation involves the lack of nanoparticle tracking in vivo. Since we did not label the SiO_2_ nanoparticles with fluorescent dyes, we were unable to assess their tissue distribution. However, prior studies have demonstrated that dye-doped silica nanoparticles enable non-invasive, real-time imaging with strong photostability and high uptake efficiency. Wang et al., 2013 [49] demonstrated that fluorescent silica nanoparticles enable in vivo tracking with enhanced photostability and multiplexing capability. Son et al., 2024 [50] developed cyanine dye-doped silica nanoparticles with tunable emission spectra, high cellular uptake, and long-term imaging potential. Ehrhorn et al., 2025 [51] synthesized near-infrared dye-labeled silica nanoparticles that showed excellent intracellular uptake and photostability, reinforcing their potential for in vivo imaging applications. Future studies should consider incorporating such fluorescently labeled nanoparticles to visualize tissue accumulation directly and determine whether NAC influences their uptake or distribution.

Finally, oxidative stress markers were measured only once, immediately after behavioral testing (Figure 6A–D), capturing cumulative effects but not temporal dynamics. Multiple time-point measurements would provide insight into fluctuations in enzyme activity and oxidative damage during exposure.

## 5. Conclusions

Exposure to 500 µg/mL SiO_2_NPs induced significant neurobehavioral alterations in adult zebrafish, including reduced general activity, increased immobility, disrupted color preference, and altered social interaction patterns. These effects were accompanied by elevated oxidative stress markers (SOD, CAT, MDA), confirming a pro-oxidant cellular environment.

NAC co-treatment alleviated several locomotor impairments and reduced oxidative stress marker levels, indicating its antioxidant and neuroprotective capacities under the tested conditions. However, NAC was less effective in improving social interaction outcomes, suggesting that its efficacy may depend on the complexity of the behavior.

In the social test, exposure to 500 µg/mL SiO_2_NPs significantly increased total distance moved and velocity, indicating anxiety-like behavior. Co-treatment with NAC effectively attenuated this increase, restoring behavior to control levels, further effectively attenuated this increase, restoring behavior to control levels, further supporting its antioxidant and neuroprotective role. Interestingly, in the same test, NAC alone did not alter social behavior under these conditions. However, its co-administration with SiO_2_NPs did not alter behavior and only partially reversed social impairments caused by nanoparticles.

## Data Availability

The original contributions presented in this study are included in the article. Further inquiries can be directed to the corresponding author.
